# Validation of the Lymphedema Quality of Life Inventory (LyQLI) in Greek Breast Cancer Patients: An Anatomical and Physiological Approach to Patient-Reported Prognosis

**DOI:** 10.7759/cureus.103257

**Published:** 2026-02-09

**Authors:** Irene Katsika, Evangelos Dimakakos, Margarita G Toumanidou, Alexandra Koreli, Ioannis Papapanagiotou, Dimitrios Nikas, Alexandros Manthas, Theodoros Piperos, Dimitrios Vergados, Pavlos Myrianthefs, Theodoros Mariolis-Sapsakos

**Affiliations:** 1 Department of Physiotherapy, General Hospital of Athens "G. Gennimatas", Athens, GRC; 2 Department of Anatomy, University of West Attica, Athens, GRC; 3 Vascular Unit, University of Athens of Hospital Sotiria, Athens, GRC; 4 Center of Prevention, Diagnosis and Treatment of Lymphedema - Lymphatic Diseases for Adults and Children, Metropolitan Hospital, Athens, GRC; 5 Department of Nursing, University of West Attica, Athens, GRC; 6 Department of Obstetrics and Gynecology, National and Kapodistrian University of Athens, Athens, GRC; 7 Faculty of Nursing, National and Kapodistrian University of Athens, Athens, GRC; 8 Laboratory of Anatomy, Faculty of Nursing, National and Kapodistrian University of Athens, Athens, GRC; 9 Department of Informatics, University of Piraeus, Athens, GRC; 10 Department of Intensive Care and Pulmonology, General Oncological Hospital of Kifisia "Oi Agioi Anargyroi", Athens, GRC; 11 Department of Nursing, National and Kapodistrian University of Athens, Athens, GRC; 12 Department of Anatomy, National and Kapodistrian University of Athens, Athens, GRC; 13 Laboratory of Anatomy, Advanced Anatomical Applications, Artificial Intelligence and Experimental Surgical Research (AAAAAIES Lab), National and Kapodistrian University of Athens, Athens, GRC

**Keywords:** anatomy, breast cancer, lymphedema, lyqli, physiology, quality of life, questionnaire validation

## Abstract

Introduction

Lymphedema has been shown to have a substantial impact on patients’ quality of life. A variety of questionnaires have been used in the literature to assess quality of life among breast cancer patients with lymphedema.

Aim

The aim of this study was to validate the Greek version of the Lymphedema Quality of Life Inventory (LyQLI), and to assess its reliability and validity among patients with lymphedema in Greece.

Materials and methods

A total of 93 patients with upper-limb lymphedema following breast cancer treatment were recruited from the General Hospital of Athens, "G. Gennimatas," Athens, Greece. The LyQLI questionnaire was translated into Greek and subsequently back-translated into English, following standard procedures for cross-cultural adaptation of patient-reported outcome measures. Content, face, and construct validity were evaluated, with the RAND version of the Short Form-36 (SF-36) serving as a comparative instrument. Reliability was assessed through test-retest analysis, internal consistency was measured using Cronbach’s alpha, and exploratory factor analysis (EFA) was conducted to examine the instrument’s underlying structure.

Results

Content and face validity analyses confirmed that the Greek version of the LyQLI is clear, concise, and user-friendly for patients with lymphedema. Construct validity was robust, with all Cronbach’s alpha coefficients exceeding 0.70 across four identified factors. Among these, the “Function” factor demonstrated the greatest impact on quality of life. Convergent validity was excellent, as LyQLI factor scores showed significant correlations with the corresponding SF-36 scales. Test-retest reliability indicated high reproducibility, and correlations between LyQLI and SF-36 scales were consistently strong (Pearson’s r > 0.3).

Conclusion

The Greek version of the LyQLI demonstrated excellent validity and reliability, confirming that it is a concise, easily comprehensible, and clinically practical instrument for evaluating quality of life among patients with lymphedema. Its psychometric properties support its use in both clinical practice and research settings, for the assessment of patient-reported outcomes in Greek-speaking populations.

## Introduction

Advancements in cancer management have substantially increased survival rates, leading to a rising number of long-term survivors [1]. Although five-year survival rates have improved significantly, these achievements are not without adverse effects [2]. Lymphedema is one of the most common side effects following cancer treatment, particularly after lymph node dissection [3]. Morgan et al. reported that approximately 25% of cancer patients develop treatment-related lymphedema [4]. Lymphedema is defined as a chronic swelling of one or more parts of the body, resulting from an impaired lymph transport capacity, due either to dysfunction or malformation of the lymphatic system [5]. The resulting mechanical insufficiency leads to fluid accumulation within the interstitial tissue spaces [5,6].

Lymphedema is classified as primary or secondary, with cancer treatment - especially for breast cancer - being the most frequent cause of secondary lymphedema in modern societies [5-7]. Studies indicate that approximately 5%-29% of women develop lymphedema within five years following breast cancer treatment [8,9].

It is a chronic condition requiring lifelong management, including manual lymphatic drainage, compression therapy, and, in some cases, pharmacological or surgical interventions [10-12]. Lymphedema is recognized as a complex and serious condition that significantly affects patients’ quality of life, and may have profound consequences for their daily functioning, social interactions, and psychological well-being [13].

Traditionally, lymphedema was considered a relatively minor side effect; however, it has been shown to cause a range of physical symptoms - such as pain, heaviness, stiffness, discomfort, and functional limitations - along with emotional and social consequences [4,14,15].

Given that lymphedema can have a substantial impact on patients’ quality of life, accurate information regarding health-related quality of life (HRQoL) outcomes in this population is essential for evidence-based decision-making and for understanding the disease’s overall impact on cancer survivors [16].

A variety of questionnaires have been used in the literature to assess quality of life among cancer patients, many of which are generic instruments, such as the European Organization for Research and Treatment of Cancer Quality of Life Questionnaire (EORTC QLQ-C30) and its breast cancer-specific module (EORTC QLQ-BR23), the Functional Assessment of Cancer Therapy-Breast (FACT-B), the Short Form-36 (SF-36), and the Nottingham Health Profile (NHP) [13,17-20].

In recent years, there has been a growing interest in developing quality of life instruments specifically designed for patients with lymphedema. Due to the unique symptoms and challenges faced by this population, it is important to use questionnaires that are tailored to the condition [21]. Several lymphedema-specific instruments have been developed to measure disease-related quality of life, including the Upper Limb Lymphedema Quality of Life (ULLQoL), Upper Limb Lymphedema-27 (ULL-27), Lymphedema Functioning, Disability, and Health Questionnaire (Lymph-ICF), Lymphedema Symptom Intensity and Distress Survey-Arm (LSIDS-A), Lymphedema Life Impact Scale (LLIS), Quality of Life Measure for Limb Lymphedema (LYMQOL), and the Lymphedema Quality of Life Inventory (LyQLI) [22-28].

The primary objective of this study was to translate and adapt the original English version of the LyQLI into Greek. The secondary objective was to evaluate the psychometric properties of the Greek version of the LyQLI by assessing its reliability and validity in a sample of patients with lymphedema in Greece.

## Materials and methods

LyQLI scale

The LyQLI scale was developed by Klernäs et al. to assess how lymphedema affects a person’s HRQoL [28]. The original version included 188 items, which were subsequently reduced to 45. Of these, 41 items are organized into three multi-item domains: the physical domain (12 items), the psychosocial domain (16 items), and the practical domain (13 items).

The instrument is concise and easy to administer, requiring no more than six minutes to complete. Demonstrating high reliability, validity, and sensitivity to changes in a patient’s condition, the LyQLI is suitable for both clinical and research applications.

Each item evaluates the impact of lymphedema over the preceding four weeks, using a four-point Likert scale. Responses to items 1-43 are scored as follows: Not at all = 0, A little = 1, Moderate = 2, and A lot = 3. Domain scores range from 0.0 to 3.0, with higher scores indicating poorer HRQoL.

Mean scores can be interpreted as follows: mean value <1.0, low negative impact on quality of life; mean value ≥1.0 and <2.0, moderate negative impact on quality of life; mean value ≥2.0, high negative impact on quality of life; responses to items 44 and 45 are scored from 0 to 3, with higher scores indicating better HRQoL.

Short Form Survey (SF-36)

The SF-36, specifically the RAND 36-Item Health Survey, is a broadly applied, generic, multidimensional instrument used in clinical settings and scientific research to assess overall HRQoL [29,30]. The questionnaire includes 36 items designed to evaluate eight core aspects of HRQoL: namely, physical functioning, role limitations associated with physical health, bodily pain, general health perceptions, vitality, social functioning, emotional role limitations, and mental health [30].

Scale scores range from 0 to 100, with higher values reflecting a better quality of life. The Greek adaptation of the SF-36 has been shown to demonstrate satisfactory reliability and validity [31,32].

Translation and editing of the Greek version of LyQLI

Permission to translate and use the LyQLI was initially obtained from the copyright holder, following the standard forward-backward translation procedure. Two native Greek speakers - a physiotherapist with expertise in lymphedema and a healthcare professional - independently translated the English version into Greek. After consensus was reached between the translators, a back-translation into English was conducted by a healthcare professional who was a native English speaker. The final Greek version was then piloted on a group of volunteers to assess the comprehensibility of the questionnaire, and all participants reported that the questionnaire was easy to read and understand.

Validation of the Greek LyQLI

For the validation of the Greek version of the LyQLI, both validity and reliability were evaluated. Validity was examined through assessments of face validity, construct validity, convergent validity, and inter-factor correlations, while reliability was evaluated in terms of internal consistency and test-retest reliability.

Study population

The study included 93 patients diagnosed with upper-limb lymphedema following breast cancer treatment. Participants were recruited from the General Hospital of Athens, "G. Gennimatas," Athens, Greece. Inclusion criteria were age ≥18 years, diagnosis confirmed by clinical assessment, and the ability to complete the questionnaire independently.

Before participating, all individuals received comprehensive oral and written explanations about the study and signed a written informed consent form. The Institutional Review Board issued approval for this study (approval no. 360; dated 8/7/2021). At baseline, each participant completed the LyQLI and the SF-36. One week later, participants completed the LyQLI only, in order to assess test-retest reliability. Questionnaires were distributed either electronically via email or in printed form. In addition, participants completed a six-item questionnaire designed to evaluate face and content validity.

Statistical analysis

Quantitative variables were summarized using means with corresponding standard deviations, as well as medians with interquartile ranges. Categorical and ordinal variables were presented as absolute counts and relative frequencies. A confirmatory factor analysis (CFA), using the maximum likelihood method, was conducted to evaluate how well the original three-factor model fit the data. Several indices were employed to assess model fit, including the Comparative Fit Index (CFI), the Tucker-Lewis Index (TLI), the Standardized Root Mean Squared Residual (SRMR), and the Root Mean Square Error of Approximation (RMSEA) [33].

Internal consistency of each LyQLI domain was assessed using Cronbach’s alpha. Test-retest reliability was evaluated using the intraclass correlation coefficient (ICC). Construct validity was examined through exploratory factor analysis (EFA) to evaluate the underlying structure of the instrument. Intercorrelations, as well as correlations between the SF-36 and LyQLI scales, were evaluated using Pearson correlation coefficients (r).

Several guidelines exist for interpreting model fit based on these indices [34-36]. The CFI and TLI range from 0 to 1, with values close to or above 0.90 generally considered indicative of acceptable fit, and values close to or above 0.95 representing excellent fit. CFI is considered particularly suitable for model evaluation, as it accounts for sample size. RMSEA values below 0.05 indicate good fit, whereas values up to 0.08 indicate acceptable fit. Similarly, SRMR values below 0.08 are indicative of good fit.

Validity

Face and Content Validity

The degree to which respondents subjectively considered the LyQLI questionnaire to adequately capture the construct it was designed to measure was assessed using a validation questionnaire, completed during the participants’ first administration of the LyQLI. This validation instrument consisted of six questions, with response options being either dichotomous or open-ended.

Construct Validity

Construct validity was evaluated by comparing LyQLI scores with those of the SF-36, which served as the “gold standard.” Correlation coefficient values greater than 0.50 were considered indicative of acceptable (moderate to strong) construct validity. Intercorrelations among LyQLI scales, as well as correlations between SF-36 and LyQLI scales, were examined using Pearson correlation coefficients (r). The strength of the correlation was interpreted as follows: very high (>0.90), high (0.70-0.90), moderate (0.50-0.70), low (0.30-0.50), and very low (<0.30) [37].

Reliability

Internal consistency reliability was assessed using Cronbach’s α coefficient. Scales with Cronbach’s α values demonstrating reliability coefficients of 0.70 or higher were deemed acceptable. All p-values were two-sided. Statistical significance was defined as p < 0.05, and all analyses were performed using IBM SPSS Statistics for Windows, Version 27 (Released 2019; IBM Corp., Armonk, NY, USA).

Test-retest reliability was evaluated by administering the questionnaire to participants on two separate occasions, assuming no significant changes in their symptoms between assessments. The retest was conducted seven days after the initial administration. Reliability was quantified using the ICC. The strength of the ICC was categorized as poor (<0.40), moderate to good (0.40-0.75), or excellent (>0.75). A coefficient value of 0 indicated an absence of reliability, while a value of 1 reflected perfect reliability [38].

## Results

Translation

All translators agreed on the finalized version of the questionnaire. The back-translation into English showed full consistency with the original version.

Completion rates were very high for the LyQLI and SF-36 questionnaires, as 93 of the 104 participants filled out both. Data from these 93 patients (93.5% women) were analyzed, and their characteristics are summarized in Table 1. The majority of participants were over 65 years of age (29%), married (68.8%), university graduates (61.3%), and pensioners (36.6%). The mean body mass index (BMI) was 29 kg/m² (SD = 4.5 kg/m²), with 44.1% classified as overweight. Furthermore, 49.5% of participants were physically active, 79.6% had public health insurance, and 39.8% had at least one comorbidity. The most frequently reported cause of lymphedema was surgery (91.4%). Partial mastectomy had been performed in 54.8% of the sample, and lymph node dissection in 97.5%. Stage II lymphedema was observed in 70.5% of patients. Additionally, 80.6% of participants presented with edema in the arm, and 74.2% in the forearm.

**Table 1:** Sample characteristics

Variable	Category	n (%)
Gender	Women	87 (93.5)
	Men	6 (6.5)
Age (years)	18 - 25	1 (1.1)
	26 - 35	2 (2.2)
	36 - 45	12 (12.9)
	46 - 55	27 (29)
	56 - 65	24 (25.8)
	66 +	27 (29)
Family status	Unmarried	24 (25.8)
	Married	64 (68.8)
	Divorced	5 (5.4)
Educational level	Primary school	3 (3.2)
	Secondary school	33 (35.5)
	University	57 (61.3)
Occupation	Public employees	16 (17.2)
	Private employees/Workers	21 (22.6)
	Freelancers	11 (11.8)
	Household	3 (3.2)
	Pensioners	34 (36.6)
	Disability pension	2 (2.2)
	Unemployed	6 (6.5)
ΒΜΙ (kg/m^2^), mean ± SD	-	29 (4.5)
BMI levels	Normal	18 (19.4)
	Overweight	41 (44.1)
	Obese	34 (36.6)
Physically active	-	46 (49.5)
If yes, at what frequency	Once a week	9 (19.1)
	Twice a week	28 (59.6)
	More than twice a week	10 (21.3)
Insurance	Without one	1 (1.1)
	Public	74 (79.6)
	Public & Private	17 (18.3)
	Private	1 (1.1)
Comorbidities	-	37 (39.8)
Reason for lymphedema	Surgery	85 (91.4)
	Ca Treatment	58 (62.4)
	Other	2 (2.2)
Type of mastectomy	Total	38 (45.2)
	Partial	46 (54.8)
	Biopsy	14 (16.7)
Lymph node cleansing	-	77 (97.5)
Lymph nodes removed, median (IQR)	-	15 (8 - 22)
Treatment after surgery	Chemotherapy	84 (93.3)
	Radiotherapy	86 (95.6)
	Hormonotherapy	56 (62.2)
	Immunotherapy	17 (18.9)
Stage	Ι	2 (2.3)
	ΙΙ	62 (70.5)
	ΙΙΙ	24 (27.3)
Anatomical site of edema	Arm	75 (80.6)
	Forearm	69 (74.2)
	Hand	51 (54.8)
	Torso	21 (22.6)
Presence of skin sores after surgery or treatments	-	15 (16.5)
Do you have easy access to care?	-	87 (93.5)
Is the operated side your primary side?	-	51 (56.7)
Did you know about lymphedema before it occurred?	-	11 (11.8)
When did you notice the change in the site's volume after surgery?	Months later	21 (22.8)
When did you notice the change in the site's volume after surgery?	One year later	28 (30.4)
When did you notice the change in the site's volume after surgery?	Two years later	22 (23.9)
When did you notice the change in the site's volume after surgery?	More than two years later	21 (22.8)
Were you informed by your healthcare providers about lymphedema before the procedure as a possible post-operative side effect?	-	30 (33)
Were you given instructions, exercises, precautions and ways to prevent lymphedema?	-	33 (35.5)
Does the feeling of heaviness or swelling change when you remain upright for more than an hour?	-	55 (59.1)

Face and content validity

More than 90% of participants reported that the questionnaires were satisfactory in terms of ease of completion. The questions were considered clear, sufficient in number, and free from redundancy. However, it was suggested that the potential impact of lymphedema on driving could be further explored. Details of the LyQLI data are presented in Table 2. A total of 88.2% of participants indicated that the specific period they referred to when completing the questionnaire represented a typical four-week period for them, as reported in Table 2 (Item 42). 

**Table 2:** Descpription of LyQLI items LyQLI, Lymphedema Quality of Life Inventory

Item		None	A Little Bit	Somewhat	A Lot
		n (%)	n (%)	n (%)	n (%)
1	Pain/aches due to my lymphedema	29 (31.2)	42 (45.2)	16 (17.2)	6 (6.5)
2	Discomfort due to my lymphedema	7 (7.5)	39 (41.9)	30 (32.3)	17 (18.3)
3	A feeling of heaviness due to my lymphedema	2 (2.2)	33 (35.5)	33 (35.5)	25 (26.9)
4	Pins and needles/numbness due to my lymphedema	35 (37.6)	34 (36.6)	22 (23.7)	2 (2.2)
5	Burning sensation/heat due to my lymphedema	50 (53.8)	29 (31.2)	11 (11.8)	3 (3.2)
6	Swelling/tightness due to my lymphedema	1 (1.1)	31 (33.3)	29 (31.2)	32 (34.4)
7	Skin problems due to my lymphedema	63 (67.7)	19 (20.4)	9 (9.7)	2 (2.2)
8	Difficulty sleeping due to my lymphedema	48 (51.6)	29 (31.2)	14 (15.1)	2 (2.2)
9	Movement difficulties due to my lymphedema	10 (10.8)	35 (37.6)	21 (22.6)	27 (29)
10	Feeling physically aware of my lymphedema all the time	1 (1.1)	38 (40.9)	27 (29)	27 (29)
11	Feeling a loss of strength in the swollen part of my body	8 (8.6)	47 (50.5)	26 (28)	12 (12.9)
12	Infection (e.g., cellulitis, erysipelas)	68 (73.1)	18 (19.4)	6 (6.5)	1 (1.1)
13	Feelings of frustration/feeling annoyed	11 (11.8)	49 (52.7)	17 (18.3)	16 (17.2)
14	Feeling anxious about whether or not the lymphedema will get worse	4 (4.3)	54 (58.1)	12 (12.9)	23 (24.7)
15	Embarrassed by lymphedema/compression garments	36 (38.7)	32 (34.4)	15 (16.1)	10 (10.8)
16	Negative changes in how I see myself	13 (14)	49 (52.7)	21 (22.6)	10 (10.8)
17	Feeling discouraged	15 (16.1)	48 (51.6)	22 (23.7)	8 (8.6)
18	Not being able to do the things I used to enjoy	12 (12.9)	54 (58.1)	17 (18.3)	10 (10.8)
19	Concerns about when to seek medical attention	39 (41.9)	36 (38.7)	15 (16.1)	3 (3.2)
20	Paying constant attention to my condition	6 (6.5)	50 (53.8)	15 (16.1)	22 (23.7)
21	Concerns about how my lymphedema affects my existing relationships	41 (44.1)	32 (34.4)	11 (11.8)	9 (9.7)
22	Concerns about how lymphedema could affect new relationships	40 (43)	31 (33.3)	9 (9.7)	13 (14)
23	Negative changes in my feelings about intimacy/sexuality	44 (47.3)	22 (23.7)	11 (11.8)	16 (17.2)
24	Feeling uncomfortable/embarrassed while doing sports and hobbies	39 (41.9)	30 (32.3)	15 (16.1)	9 (9.7)
25	Feeling uncomfortable/embarrassed when attending social activities with friends and at work	29 (31.2)	42 (45.2)	15 (16.1)	7 (7.5)
26	Having to ask for help in different situations	38 (40.9)	35 (37.6)	16 (17.2)	4 (4.3)
27	Concerns about negative changes in my appearance	5 (5.4)	49 (52.7)	22 (23.7)	17 (18.3)
28	Having to answer questions about my lymphedema	44 (47.3)	34 (36.6)	11 (11.8)	4 (4.3)
29	Personal activities of daily living (e.g., dressing, combing hair, foot care)	11 (11.8)	48 (51.6)	25 (26.9)	9 (9.7)
30	Normal daily activities (e.g., doing housework, sports, and hobby activities)	7 (7.5)	33 (35.5)	31 (33.3)	22 (23.7)
31	Employment activities	9 (9.7)	43 (46.2)	25 (26.9)	16 (17.2)
32	Learning to do things differently	14 (15.1)	54 (58.1)	17 (18.3)	8 (8.6)
33	Having less energy to do activities (e.g., personal, normal daily or employment)	25 (26.9)	48 (51.6)	17 (18.3)	3 (3.2)
34	Financial costs of managing my lymphedema (e.g., clothes, shoes, treatments, garments)	3 (3.2)	13 (14)	35 (37.6)	42 (45.2)
35	Finding well-functioning compression garments (e.g., stockings, sleeves, gloves)	18 (19.4)	46 (49.5)	20 (21.5)	9 (9.7)
36	Traveling long distances by car, train, plane, etc.	18 (19.4)	33 (35.5)	22 (23.7)	20 (21.5)
37	Finding clothes and shoes that are comfortable and attractive, the right size, and type of material	13 (14)	49 (52.7)	14 (15.1)	17 (18.3)
38	Limitations in hot weather/sun	4 (4.3)	32 (34.4)	13 (14)	44 (47.3)
39	The constant self-care I need to do to stop my lymphedema from getting worse	2 (2.2)	51 (54.8)	18 (19.4)	22 (23.7)
40	Obtaining information about how to manage my lymphedema	41 (44.1)	38 (40.9)	7 (7.5)	7 (7.5)
41	Being prepared for emergencies (e.g., always having a script for antibiotics)	26 (28)	51 (54.8)	10 (10.8)	6 (6.5)
		Νο	Yes		
42	In terms of your lymphedema, has this been a typical four-week period for you?	11 (11.8)	82 (88.2)	-	-

Test-retest reliability

The test-retest procedure was conducted with all 93 participants, and the results are presented in Table 3. A significant level of agreement was observed between the two administrations of the questionnaire across all items.

**Table 3:** Test-retest results of LyQLI items ICC, Intraclass Correlation Coefficient; 95% CI, 95% Confidence Interval; LyQLI, Lymphedema Quality of Life Inventory

Item	ICC	95% CI	p-value
1	0.99	0.98 - 0.99	<0.001
2	0.89	0.83 - 0.92	<0.001
3	0.90	0.85 - 0.94	<0.001
4	0.87	0.81 - 0.92	<0.001
5	0.88	0.81 - 0.92	<0.001
6	0.96	0.93 - 0.97	<0.001
7	0.96	0.94 - 0.97	<0.001
8	0.94	0.90 - 0.96	<0.001
9	0.90	0.85 - 0.93	<0.001
10	0.93	0.89 - 0.95	<0.001
11	0.89	0.84 - 0.93	<0.001
12	0.97	0.95 - 0.98	<0.001
13	0.96	0.94 - 0.97	<0.001
14	0.92	0.89 - 0.95	<0.001
15	0.95	0.92 - 0.96	<0.001
16	0.96	0.94 - 0.97	<0.001
17	0.88	0.82 - 0.92	<0.001
18	0.94	0.91 - 0.96	<0.001
19	0.96	0.94 - 0.97	<0.001
20	0.95	0.92 - 0.97	<0.001
21	0.92	0.87 - 0.94	<0.001
22	0.93	0.89 - 0.95	<0.001
23	0.96	0.94 - 0.97	<0.001
24	0.93	0.90 - 0.96	<0.001
25	0.93	0.90 - 0.95	<0.001
26	0.95	0.92 - 0.96	<0.001
27	1.00	0.99 - 1.00	<0.001
28	0.97	0.96 - 0.98	<0.001
29	0.92	0.89 - 0.95	<0.001
30	0.90	0.85 - 0.93	<0.001
31	0.90	0.85 - 0.94	<0.001
32	0.94	0.91 - 0.96	<0.001
33	0.95	0.92 - 0.97	<0.001
34	0.96	0.94 - 0.97	<0.001
35	0.95	0.92 - 0.97	<0.001
36	0.94	0.91 - 0.96	<0.001
37	0.99	0.98 - 0.99	<0.001
38	0.96	0.93 - 0.97	<0.001
39	0.95	0.92 - 0.97	<0.001
40	0.99	0.98 - 0.99	<0.001
41	0.98	0.97 - 0.99	<0.001
44	0.95	0.92 - 0.97	<0.001
45	0.93	0.89 - 0.95	<0.001
	Kappa	SE	p-value
42	0.59	0.13	<0.001

Given that the data were adequate for factor analysis (Kaiser-Meyer-Olkin (KMO) measure = 0.78; Bartlett’s test of sphericity, p < 0.001), an EFA was subsequently performed. The results of the EFA are presented in Table 4.

**Table 4:** EFA results of LyQLI scale EFA, Exploratory Factor Analysis; LyQLI, Lymphedema Quality of Life Inventory

Item	Mood	Appearance and Body Image	Function	Symptoms
5	0.41	-	-	-
7	0.59	-	-	-
12	0.57	-	-	-
14	0.70	-	-	-
19	0.53	-	-	-
20	0.50	-	-	-
26	0.42	-	-	-
39	0.59	-	-	-
40	0.46	-	-	-
41	0.70	-	-	-
21	-	0.74	-	-
22	-	0.79	-	-
23	-	0.73	-	-
24	-	0.66	-	-
25	-	0.82	-	-
27	-	0.69	-	-
28	-	0.76	-	-
13	-	0.60	-	-
15	-	0.68	-	-
16	-	0.76	-	-
17	-	0.68	-	-
18	-	-	0.46	-
29	-	-	0.66	-
30	-	-	0.74	-
31	-	-	0.74	-
32	-	-	0.60	-
33	-	-	0.53	-
35	-	-	0.54	-
36	-	-	0.46	-
37	-	-	0.45	-
38	-	-	0.49	-
1	-	-	-	0.58
2	-	-	-	0.77
3	-	-	-	0.60
4	-	-	-	0.44
6	-	-	-	0.63
8	-	-	-	0.50
9	-	-	-	0.62
11	-	-	-	0.46
% Variance explained	28.0	13.5	6.4	4.6

Factor analysis

The analysis identified four factors, which together accounted for 52.5% of the overall variance. Specifically, the first factor (“Mood”) consisted of 10 items and accounted for 28% of the variance. The second factor (“Appearance and Body Image”) comprised 11 items and explained 13.5% of the variance. The third factor (“Function”) included 10 items and explained 6.4% of the variance, while the fourth factor (“Symptoms”) consisted of eight items and explained 4.6% of the variance. Two items (items 10 and 34) had loadings below 0.40 and were therefore not included in any of the factors.

All four factors that emerged from the EFA demonstrated acceptable reliability, as all Cronbach’s alpha coefficients exceeded 0.70 (Table 5). The removal of any single item did not substantially improve the reliability of its respective factor; therefore, no items were removed.

**Table 5:** Reliability analysis of LyQLI items LyQLI, Lymphedema Quality of Life Inventory

Item	Corrected Item - Total Correlation	Cronbach's Alpha If Item Deleted	Cronbach's Alpha
5	0.48	0.83	0.84
7	0.55	0.83	
12	0.46	0.84	
14	0.64	0.82	
19	0.59	0.82	
20	0.51	0.83	
26	0.42	0.84	
39	0.64	0.82	
40	0.54	0.83	
41	0.57	0.83	
13	0.64	0.91	0.92
15	0.57	0.91	
16	0.68	0.91	
17	0.61	0.91	
21	0.73	0.90	
22	0.77	0.90	
23	0.69	0.91	
24	0.60	0.91	
25	0.77	0.90	
27	0.69	0.91	
28	0.67	0.91	
18	0.66	0.86	0.88
29	0.57	0.87	
30	0.68	0.86	
31	0.58	0.87	
32	0.59	0.87	
33	0.64	0.86	
35	0.61	0.87	
36	0.52	0.87	
37	0.58	0.87	
38	0.63	0.86	
1	0.63	0.83	0.85
2	0.54	0.84	
3	0.64	0.83	
4	0.46	0.85	
6	0.62	0.83	
8	0.57	0.84	
9	0.66	0.82	
11	0.60	0.83	

The mean score for the “Mood” factor was 0.97 (SD = 0.54), for the “Appearance and Body Image” factor, 1.09 (SD = 0.69), for the “Function” factor, 1.42 (SD = 0.61), and for the “Symptoms” factor, 1.40 (SD = 0.61), as presented in Table 6.

**Table 6:** Mean ± SD and Pearson’s correlations among LyQLI factors **p < 0.01; ***p < 0.001 LyQLI, Lymphedema Quality of Life Inventory

Factor		Mean ± SD	Pearson's Correlation Coefficients			
			1	2	3	4
1.	Mood	0.97 (0.54)	1.00	0.42***	0.61***	0.56***
2.	Appearance and body image	1.09 (0.69)	-	1.00	0.27**	0.20
3.	Function	1.42 (0.61)	-	-	1.00	0.63***
4.	Symptoms	1.40 (0.61)	-	-	-	1.00

**Figure 1:**
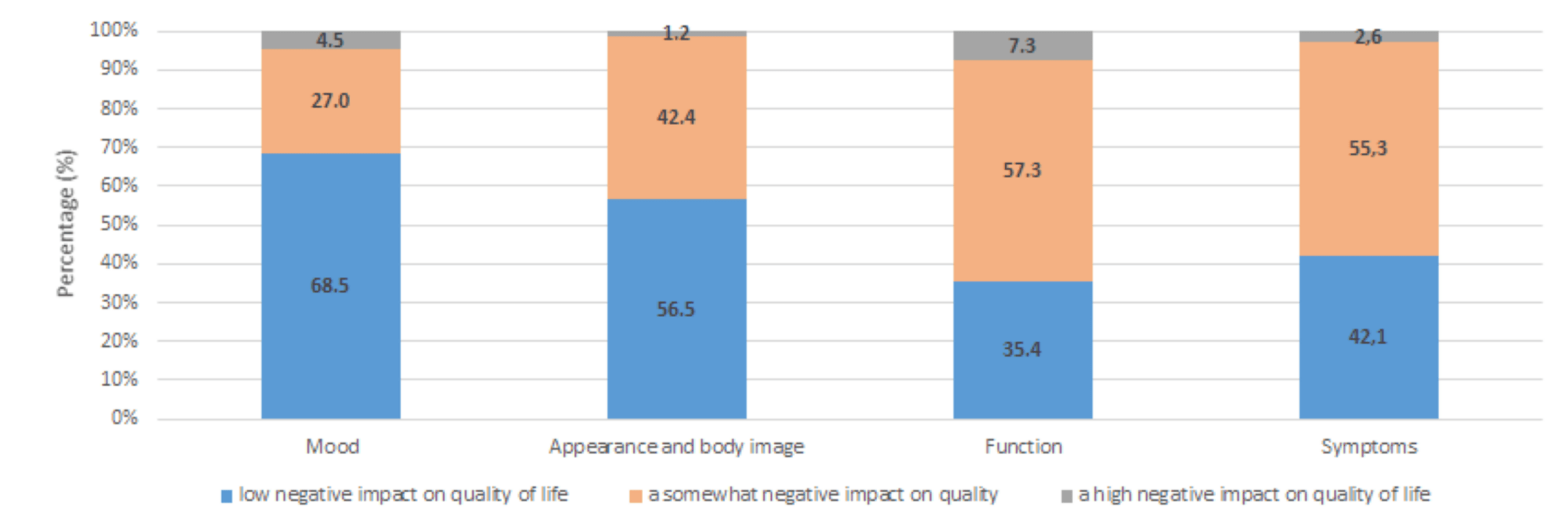
Negative impact of LyQLI factors on patients' quality of life LyQLI, Lymphedema Quality of Life Inventory

All factors were significantly correlated with each other (p < 0.001), except for the “Symptoms” and “Appearance and Body Image” factors, which were not significantly correlated (p > 0.5). The level of each factor’s negative impact on patients’ QoL is depicted in Figure 1. The “Function” factor has the greatest percentage of participants with a high negative impact on their quality of life (7.3%), followed by the “Mood” factor (4.5%), even though this factor has the lowest negative impact overall.

Regarding convergent validity, the LyQLI factors were correlated with the SF-36 scales (Table 7). Significant negative correlations were observed across almost all SF-36 and LyQLI scores, indicating that a higher quality of life as measured by one instrument was significantly associated with a lower quality of life as measured by the other instrument.

**Table 7:** Descriptive statistics of SF-36 scales and correlation with LyQLI factors LyQLI, Lymphedema Quality of Life Inventory; SF-36, Short Form-36

SF-36 Domains	Statistic	Mood	Appearance and Body Image	Function	Symptoms
Physical functioning, Mean ± SD = 53 (21.8)	r	-0.32	0.04	-0.51	-0.43
	p	2	702	<0.001	<0.001
Physical role, Mean ± SD = 41.9 (43.0)	r	-0.09	0.16	-0.31	-0.36
	p	399	125	3	<0.001
Physical pain, Mean ± SD = 76.1 (19.5)	r	-0.34	-0.07	-0.32	-0.45
	p	1	504	2	<0.001
General health, Mean ± SD = 59.6 (14.5)	r	-0.35	-0.20	-0.37	-0.38
	p	1	53	<0.001	<0.001
Vitality, Mean ± SD = 51.5 (12.8)	r	-0.35	-0.19	-0.44	-0.49
	p	1	64	<0.001	<0.001
Social role, Mean ± SD = 72.2 (18.3)	r	-0.43	-0.28	-0.48	-0.48
	p	<0.001	7	<0.001	<0.001
Emotional role, Mean ± SD = 65.2 (42.8)	r	-0.21	-0.01	-0.27	-0.24
	p	40	898	8	18
Mental health, Mean ± SD = 63 (11.9)	r	-0.39	-0.35	-0.21	-0.32
	p	<0.001	1	42	2

## Discussion

Since no validated Greek lymphedema-specific questionnaires existed at the time of data collection, the current study sought to translate the LyQLI into Greek and examine its validity and reliability.

The LyQLI was successfully translated into Greek, and the findings suggest that the Greek version of the LyQLI demonstrates satisfactory reliability and validity within the studied population. The Greek LyQLI exhibited high construct validity, significant convergent validity, and good internal consistency. Four factors emerged from the factor analysis, explaining 52.5% of the total variance.

In this study, the reliability and validity of the LyQLI were tested among Greek patients with upper-limb lymphedema. The established SF-36 questionnaire was selected as the reference instrument for the validity analysis, as it is a widely used and well-validated measure of general HRQoL, both in Greece and internationally [31,39].

For construct validity, CFA was initially performed; however, the structure reported in the literature was not confirmed. Consequently, EFA was conducted, revealing four distinct factors. The correlations between these factors were found to be strong and statistically significant (p < 0.001), except for the factors “Symptoms” and “Appearance and Body Image,” which were not significantly correlated (p > 0.5). This finding may be explained by the fact that the majority of participants were over 65 years of age and may prioritize survival over body image concerns. Additionally, most patients completed the questionnaire during a typical period without symptom exacerbation.

The “Function” and “Mood” factors had the greatest negative impact on patients’ quality of life (7.3% and 4.5%, respectively). A large proportion of participants (44.1%) were overweight, which may account for the greater functional burden of lymphedema on their daily activities.

Potential confounding factors, such as age, BMI, and disease duration, were not fully controlled and may have influenced the observed associations. In addition, the predominance of older female participants may have affected certain domain scores, particularly those related to body image and symptoms.

Regarding convergent validity, the LyQLI factors were correlated with the SF-36 subscales. Significant negative correlations were observed across almost all SF-36 and LyQLI scores, indicating that a higher quality of life, as measured by one instrument, was significantly associated with a higher quality of life, as measured by the other instrument.

The Greek LyQLI demonstrated satisfactory psychometric properties in this sample of patients with upper-limb lymphedema, including high internal consistency across all items and acceptable reliability for the four factors identified through EFA, with Cronbach’s alpha coefficients exceeding 0.70. Test-retest analysis further supported the stability of the instrument, showing excellent agreement between administrations (ICC > 0.90, p < 0.001). These findings are consistent with previous validation studies in other countries. For instance, the Swedish version of the LyQLI also demonstrated good internal consistency and test-retest reliability, supporting the robustness of the instrument across cultural contexts [40]. Similarly, the original Australian version confirmed the utility of the LyQLI in capturing the impact of lymphedema on multiple aspects of quality of life [28].

Despite these encouraging results, several limitations should be considered. First, the study sample was relatively small and predominantly female (87 women and 6 men), reflecting the focus on breast cancer-related lymphedema. This limits the generalizability of the findings to male patients or to lymphedema of other etiologies, but the results remain robust for the primary target population.

Second, although the majority of participants reported that the referenced period reflected a typical four-week interval, a small proportion did not, which may affect the accuracy of responses. Additionally, certain aspects of daily life, such as the effect of lymphedema on driving or more physically demanding activities, were not fully explored, suggesting that the LyQLI may not capture all dimensions of patient experience.

Finally, the assumptions underlying the theoretical framework and analytical models were not extensively tested, which could affect the generalizability of the results to other populations or contexts.

Nevertheless, these limitations provide clear guidance for future research, including larger, more diverse samples and a more comprehensive assessment of daily activities, which could further validate and expand the applicability of the findings. Additionally, simplifying the instrument or further evaluating underexplored domains may enhance its usability and comprehensiveness for clinical and research purposes. Overall, these findings indicate that the Greek LyQLI is a reliable and valid tool for assessing quality of life in patients with breast cancer-related lymphedema.

## Conclusions

The Greek version of the LyQLI questionnaire constitutes a specific instrument for assessing the quality of life in patients with lymphedema and is appropriate for use in the Greek population. It is a brief and easily comprehensible questionnaire. The findings indicate that the instrument is both valid and reliable, and it is recommended for evaluating the quality of life of patients with lymphedema.
